# Transcriptomics Analysis Reveals Shared Pathways in Peripheral Blood Mononuclear Cells and Brain Tissues of Patients With Schizophrenia

**DOI:** 10.3389/fpsyt.2021.716722

**Published:** 2021-09-22

**Authors:** Xuemian Song, Yiyun Liu, Juncai Pu, Siwen Gui, Xiaogang Zhong, Xiaopeng Chen, Weiyi Chen, Xiang Chen, Yue Chen, Haiyang Wang, Ke Cheng, Libo Zhao, Peng Xie

**Affiliations:** ^1^NHC Key Laboratory of Diagnosis and Treatment on Brain Functional Diseases, The First Affiliated Hospital of Chongqing Medical University, Chongqing, China; ^2^State Key Laboratory of Ultrasound in Medicine and Engineering, Chongqing Medical University, Chongqing, China; ^3^Key Laboratory of Psychoseomadsy, Stomatological Hospital of Chongqing Medical University, Chongqing, China; ^4^Department of Neurology, Yongchuan Hospital, Chongqing Medical University, Chongqing, China

**Keywords:** schizophrenia, transcriptomics, peripheral blood mononuclear cells, phospholipid metabolism, ribosome signal transduction, mitochondrial dysfunction

## Abstract

**Background:** Schizophrenia is a serious mental disorder with complicated biological mechanisms. Few studies explore the transcriptional features that are shared in brain tissue and peripheral blood. In the present study, we aimed to explore the biological pathways with similar expression patterns in both peripheral blood mononuclear cells (PBMCs) and brain tissues.

**Methods:** The present study used transcriptomics technology to detect mRNA expression of PBMCs of 10 drug-naïve patients with schizophrenia and 20 healthy controls. Transcriptome data sets of brain tissue of patients with schizophrenia downloaded from public databases were also analyzed in our study. The biological pathways with similar expression patterns in the PBMCs and brain tissues were uncovered by differential expression analysis, weighted gene co-expression network analysis (WGCNA), and pathway analysis. Finally, the expression levels of differential expressed genes (DEGs) were validated by real-time fluorescence quantitative polymerase chain reaction (qPCR) in another 12 drug-naïve patients with schizophrenia and 12 healthy controls.

**Results:** We identified 542 DEGs, 51 DEGs, 732 DEGs, and 104 DEGs in PBMCs, dorsolateral prefrontal cortex, anterior cingulate gyrus, and nucleus accumbent, respectively. Five DEG clusters were recognized as having similar gene expression patterns in PBMCs and brain tissues by WGCNA. The pathway analysis illustrates that these DEG clusters are mainly enriched in several biological pathways that are related to phospholipid metabolism, ribosome signal transduction, and mitochondrial oxidative phosphorylation. The differential significance of *PLAAT3, PLAAT4, PLD2, RPS29, RPL30, COX7C, COX7A2, NDUFAF2*, and *ATP5ME* were confirmed by qPCR.

**Conclusions:** This study finds that the pathways associated with phospholipid metabolism, ribosome signal transduction, and energy metabolism have similar expression patterns in PBMCs and brain tissues of patients with schizophrenia. Our results supply a novel insight for revealing the pathogenesis of schizophrenia and might offer a new approach to explore potential biological markers of peripheral blood in schizophrenia.

## Introduction

Schizophrenia is a serious mental disorder characterized by hallucinations and delusions (1) with a lifetime risk of 0.72% (2), which brings a huge burden to more than 20 million patients and their families worldwide (3). The viewpoint that schizophrenia is characterized by alteration of multiple genes rather than a single gene or pathway is confirmed in the etiology study (4). However, the precise mechanism of schizophrenia is still unclear.

Molecular alterations in the central nervous system (CNS) are considered to be the important feature of schizophrenia. Transcriptomics analysis contributing to understanding the changes of upstream regulation sequences has been used to uncover the potential pathophysiologic mechanism of schizophrenia in the brain tissues (5–7). However, the applications of transcriptomics analysis in the CNS have been restricted by the difficulty in collecting postmortem brain tissue. In contrast, peripheral blood mononuclear cells (PBMCs) become an important investigation carrier of schizophrenia because they can be acquired easily from patients and partly reflect the molecular alterations in the CNS (8, 9). PBMCs can selectively migrate and infiltrate into the brain and then participate in a series of pathological reactions after crossing the blood–brain barrier (10). Researchers demonstrate that PBMCs show similar gene expression patterns to the brain (11–13). Furthermore, some studies demonstrate impaired glycolytic response (9) and altered neural signaling and immune pathways (14) in PBMCs of patients with schizophrenia. However, it is still unclear which gene expression patterns and biological pathways in the brain are reflected by PBMCs in schizophrenia. Thus, we present a hypothesis that PBMCs could reflect the molecular alterations in brain tissues of patients with schizophrenia to a certain extent and be a valid surrogate to explore molecular alterations in schizophrenia.

In the present study, we aimed to identify transcriptional alterations in PBMCs of drug-naïve patients with schizophrenia and to explore the biological pathways with similar expression patterns in both PBMCs and brain tissues. To this end, we compared the transcriptome profiles of PBMCs between patients with schizophrenia and healthy controls. We also reanalyzed the transcriptome profiles of dorsolateral prefrontal cortex (DLPFC), anterior cingulate gyrus (AnCg), and nucleus accumbent (nAcc) using public data sets from another research team and identified differential expressed genes (DEGs) in these data sets. Transcriptional clusters with similar expression patterns between PBMCs and brain tissues were revealed by weighted gene co-expression network analysis (WGCNA), and the underlying biological functions of these clusters were identified using pathway analysis. Relative expression of DEGs in these pathways were further validated by real-time quantitative polymerase chain reaction (qPCR). Our study supplies a novel perspective for revealing the potential pathogenesis of schizophrenia.

## Methods and Materials

### Participants

A total of 22 adult drug-naïve patients with schizophrenia were recruited from the psychiatric center in the First Affiliated Hospital of Chongqing Medical University based on the following inclusion criteria: (1) conforming to the diagnostic criteria for schizophrenia based on the *Diagnostic and Statistical Manual of Mental Disorders*, fourth edition, text revision (15); (2) without other neurological disorders, mood disorders, substance dependence, or serious physical illness; and (3) female patients were not pregnant, lactating, or menstruating. On the other hand, the participants with the following characteristics were excluded: (1) complicating with other neuropsychiatric diseases, (2) complicating with serious somatopathy, and (3) drug abuse and substance dependence. The severity of disease was assessed by the positive and negative syndrome scale (PANSS). Meanwhile, 32 healthy controls matched with the patients in age, gender, and body mass index (BMI) were recruited from the medical examination center in the First Affiliated Hospital of Chongqing Medical University. No healthy controls had a past or family history of mental illness. We divided all subjects into two cohorts. PBMC samples from 10 patients and 20 healthy controls were used for transcriptomics analysis, and the PBMC samples from the remaining 12 patients and 12 healthy controls were used for qPCR validation.

The present study was performed according to the protocol approved by the Ethical Committee of Chongqing Medical University and in accordance with the Helsinki Declaration for all experimentations. All participants signed the informed consent for their participation before any procedure was carried out.

### Sample Preparation

The whole blood samples were collected into EDTA-coated tubes (BD Vacutainer®, Franklin Lakes, NJ, USA) and were diluted by an equal volume of phosphate-buffered saline (PBS) (YuanPei, China). PBMCs were isolated by Ficoll-Paque Plus (GE Healthcare Bio-sciences AB, Sweden). Briefly, the diluted blood was overlaid onto Ficoll-Paque Plus. PBMCs were obtained after centrifugation (2,000 rpm, 20 min) and further purified by washing three times in PBS. These PBMCs samples were stored at −80°C prior to sequencing.

### RNA Sequencing

#### RNA Extraction and Quality Evaluation

Total RNA was extracted by the miRNeasy Mini kit (Qiagen, Germany) and quantitatively detected by Quant-iT^TM^ RiboGreen (Thermo Fisher Scientific, USA). RNA was subsequently electrophoresed using a 1% agarose gel to assess the integrity and the presence or absence of DNA contamination. The purity of RNA was assessed by NanoPhotometer spectrophotometer (Implen GmbH, Germany). At last, RNA integrity was precisely measured by Agilent 2100 Bioanalyzer system (Agilent Technologies, USA).

#### cDNA Libraries Construction

cDNA libraries were constructed using the NEBNext® Ultra^TM^ RNA Library Prep Kit (Illumina, USA) following the manufacturer's instructions. Briefly, 1 μg total RNA was used for each sample. mRNA was purified by magnetic beads with oligo (dT) and fragmented by divalent cation in the NEB fragmentation buffer (NEB, USA). The first strand of cDNA was synthesized by using mRNA fragments as templates and random oligonucleotides as primers in the M-Mulv reverse transcriptase system. The second strand was synthesized with DNA Polymerase I. Then, the eligible cDNAs (around 200 bp) were screened using the AMPure XP system (Beckman Coulter, USA) and amplified by PCR. Finally, we obtained the cDNA libraries after purification again.

cDNA libraries were preliminarily quantified through Qubit2.0 Fluorometer (Thermo Fisher SCIENTIFIC, USA). Subsequently, the libraries were diluted to 1.5 ng/μl for detection of inter size using Agilent 2100 bioanalyzer (Agilent, USA). Accurate examination of the concentration of cDNA libraries were performed by qPCR when the inter size was eligible. To guarantee the availability of cDNA libraries, we required the concentration of cDNA libraries to be >2 nM.

#### Sequencing Procedures

DNA polymerase, primers, four different types of dNTP containing fluorescent markers, and cDNA libraries were mixed into the flow cell. The sequence information of the mRNA fragments was acquired by converting the fluorescence signals captured by the sequenator (Illumina NovaSeq6000, USA) into sequencing peaks. To obtain clean reads, raw reads with Qphred ≤ 20, adapters, or unknown base-pairs were removed.

### Acquisition of Transcriptome Data From the Brain of Patients With Schizophrenia

To survey the interrelation of gene expression patterns between PBMCs and brain tissue, we retrieved transcriptome data on the brain of patients with schizophrenia in the Gene Expression Omnibus (GEO, https://www.ncbi.nlm.nih.gov/gds). We selected the data set with accession number GSE80655 (16) because this data set contained transcriptome data of three brain regions (DLPFC, AnCg, and nAcc) that are highly associated with schizophrenia (17, 18). Raw transcriptome data in fastq format from the brain of patients with schizophrenia was downloaded from GEO. This data set included transcriptome data from DLPFC of 22 patients and 16 healthy controls, AnCg of 15 patients and 11 healthy controls, and nAcc of 13 patients and 18 healthy controls (Supplementary Table 1).

### Data Processing

#### Data Preprocessing and Quality Control

The consistent matching process was performed in the transcriptome data from PBMC and brain tissues. Briefly, the files of transcriptome in fastq format were matched to human reference genome and gene model annotation files (Homo_sapiens_Ensemble_90) by HISAT2 (v2.0.5). The counts of genes were calculated and standardized into fragments per kilobase of exon model per million mapped fragments (FPKM) by StringTie (v1.3.3.b). We kept genes with FPKM > 1 in more than 80% of samples for further analysis; log-transformed FPKMs were used for drawing and network analysis. The sample outliers were evaluated by calculating standardized sample connectivity (*Z.K*), and the samples with *Z.K* < −2 were eliminated (19).

#### Differential Expression Analysis

For differential expression analysis, the R software v3.6.1 and *edgeR* statistical package (v3.26.8) were used. Genes with *p* < 0.05 and fold changes (FCs) > 1.3 were considered as DEGs (20). The heat map was plotted using the *pheatmap* package (v1.0.12). The Venn diagram of shared DEGs among the four datasets was drew using the *VennDiagram* package (v1.6.20).

#### Weighted Gene Co-expression Network Analysis

WGCNA is conducive to discover a set of genes of interest through describing the correlation patterns among genes across transcriptome samples. The methods of co-expression network construction and module statistics in each data set were generally consistent with the approach in Langfelder et al. (21). In brief, the transcriptional data in each data set was applied to construct a co-expression network by the *WGCNA* package (v1.96) in the R software. A signed weighted adjacency matrix was generated by calculating the pairwise Pearson correlation matrix between genes and converting it using a power adjacency function. The soft-threshold power β was chosen to satisfy the scale-free topology criterion when *R*^2^ was approximated to 0.80. The adjacency matrix then was transformed into a topological overlap matrix (TOM) to evaluate the extent of dissimilarity between genes pairs. Finally, 1-TOM was used as the distance to construct the hierarchical clustering. The minimum module size was set at 30. Each module was named with a separate color.

To measure the similarities of expression patterns between PBMCs and the three brain regions, we estimated the preservation of brain tissue modules in the network of PBMCs. The process of discovering preserved modules was nearly consistent with the method in Langfelder et al. (22). In brief, to survey the gene expression patterns of which brain modules could be reflected by PBMCs, the co-expression network of each brain tissue was set as reference data, respectively, and the co-expression network of PBMCs was set as test data. The degree of preservation of each gene module in the brain tissue was evaluated by the *Zsummary*, which was calculated by the *clusterRepro* package (v0.9) (23) in the R software. The following thresholds were used for determining the extent of preservation: if *Zsummary* <2, it denotes without preservation; if 2 < *Zsummary* <10, it denotes weak-to-moderate preservation; if *Zsummary* > 10, it denotes high preservation.

To search which DEGs in PBMCs highly overlapped with the gene modules of the brain tissue, the DEGs in PBMCs that overlapped with co-expression networks in the brain tissues were identified using a one-sided Fisher's exact test. The DEGs in PBMCs with a false discovery rate (FDR) < 0.05 were deemed to significantly overlap with the gene modules of brain tissue.

#### Pathway Analysis

To expound the pathways with similar expression patterns in both PBMCs and brain tissues, the DEGs in PBMCs that significantly enriched in the any brain modules with *Zsummary* > 2 were used for pathway analysis. The pathway analyses were performed using Ingenuity Pathway Analysis (IPA, http://www.ingenuity.com). The pathways were adjusted by Benjamini–Hochberg (*p*-value < 0.05).

### Real-Time Quantitative Polymerase Chain Reaction

Total mRNA from PMBCs was extracted using Trizol (Invitrogen, USA) and reverse-transcribed into cDNA using a PrimeScript®RT Reagent Kit (Takara, Japan). qPCR reactions were performed in SYBR®Premix Ex Taq™II (Takara, Japan) on a LightCyler 96 System (Roche, Switzerland). The amplification protocol includes 95°C for 30 s and 40 cycles: 95°C for 5 s and 60°C for 30 s. The quantified examination of gene expression was performed by the comparative Ct method (2^−ΔΔCt^). The primers (Table 1) were designed using BLAST (https://blast.ncbi.nlm.nih.gov/Blast.cgi) and purchased from Sangon Biotech (Shanghai, China). *GAPDH* was used as an internal standard for normalizing target genes.

**Table 1:** Primer sequences used for qPCR analysis.

**Gene names**	**Forward primer**	**Reverse primer**
*PLAAT3*	GTCCGCCCTGACTG ACAAG	GCTCGCAGTTCT CACTGGT
*PLAAT4*	TGGACCATGAGTAC CAACCAC	GCGGGACTTGCCAT ATCTCAG
*PLD2*	CAGATGGAGTCCG ATGAGGTG	CCGCTGGTATATCT TTCGGTG
*PLCB3*	TTGAGCGGTTCCT GAACAAG	CACTTCGTTGAGTC TCGGGT
*CHKA*	ATTACAGGGGATTCGA CATTGGA	GCTGTTGTTTCTTGG TGGGAT
*RPS29*	CGAAAATTCGGCCA GGGTTC	TGCCAAGGAAGACA GCTCAG
*RPS27*	TGCCTCTCGCAAA GGATCTC	TGTGCATGGCTAAA GACCGT
*RPL9*	GCACAGTTATCGTG AAGGGC	TTACCCCACCATTTG TCAACC
*RPL35A*	AAAGGGAGCACA CAGCTCTT	GGTTTTGTTTGGTT TGCCGC
*RPL24*	GAGCTGTGCAGTT TTAGCGG	CTGGAATTTGACTG CTCGGC
*RPL30*	AAAGTGGGAAGTAC GTCCTGG	GCAGTTGTTAGCGAG AATGACC
*COX7C*	CACAACCTCTGTGG TCCGTAG	GGTGTAGCAAATGCAG ATCCAAA
*COX7A2*	GGAATCTGCTGGC TCTTCGT	TGGCTCTATACAGGA GGGCA
*NDUFAF2*	AAGGGAAGTGAAGG AGCACG	CCAAGCTTCCCAT TCTGTTGG
*NDUFA1*	GCGTACATCCACAG GTTCAC	ACTCCAGAGATGCG CCTATC
*ATP5F1E*	CGAGCTCCGCTT TCGCTA	GGGAGTATCGGATGTA GCTGAG
*ATP5ME*	GAGCCACGCGCTA CAATTAC	GCCAATTCTCTGGC AATCCG
*GAPDH*	GGAGCGAGATCCCT CCAAAAT	GGCTGTTGTCATACTT CTCATGG

### Statistics

All statistical analyses of demographic variables were performed using R software v3.6.1. Normality was assessed by Shapiro–Wilk test, continuous data with normal distribution was compared by Student's *t-*test, and continuous data with non-normal distribution was compared by Mann–Whitney *U* test. The sex difference was analyzed by Fisher's exact test. A *p*-value < 0.05 was considered to be statistically significant. The effect size (Cohen's *d*) based on the formula and the kurtosis and skewness of variables were calculated by the moments package in the R software.

## Results

### Demographic Characteristics

A total of 22 drug-naïve patients with schizophrenia and 32 healthy controls were recruited in our study. The demographic data, including age, gender, and BMI, was similarly distributed between patients and healthy controls in the two cohorts, respectively (Table 2). There were no statistical differences in the PANSS scores and duration of illness of patients with schizophrenia between the two cohorts. The effective size, kurtosis, and skewness of variables are displayed in Supplementary Table 2.

**Table 2:** The clinical characteristics of the subjects.

	**Cohort 1**				**Cohort 2**			
**Variables**	**SCZ**	**HC**	***P*-values**	***t*/*Z***	**SCZ**	**HC**	***P*-values**	***t***
Sample size	10	20	–		12	12	–	
Sex (M/F)	5/5	10/10	1.00		5/7	4/8	1.00	
Age (years)	46.00 ± 12.53	48.00 ± 9.24	0.66	0.45^a^	44.17 ± 12.72	48.08 ± 5.02	0.34	0.99^a^
BMI (kg/m^2^)	22.21 ± 2.88	22.02 ± 5.20	0.52	1.04^b^	23.84 ± 4.19	24.80 ± 2.97	0.52	0.65^a^
PANSS total score	112.00 ± 35.14	–	–		111.50 ± 20.16	–	–	
Duration of illness (months)	29.73 ± 74.17	–	–		27.25 ± 27.75	–	–	

*HC, healthy controls; SCZ, schizophrenia; BMI, body mass index; PANSS, positive and negative syndrome scale. Values were expressed as mean ± standard deviation.*

a *The value of the Student's t test,*

b *the value of the Mann-Whitney test*.

### Overview of Gene Expression Profiling

A total of 58,302 and 60,617 transcripts were identified in the transcriptomics analysis of PBMCs and brain tissues, respectively. Then 8,911 shared transcripts meeting the criterion of FPKM > 1 in more than 80% of samples in the PBMC, DLPFC, AnCg, and nAcc data sets were reserved for following analysis. Based on the standardized sample connectivity, 2, 1, 2, and 1 outlier samples were excluded from the PBMC, DLPFC, AnCg, and nAcc data sets, respectively (Supplementary Figure 1).

### Differential Expression Analysis

**Figure 1:**
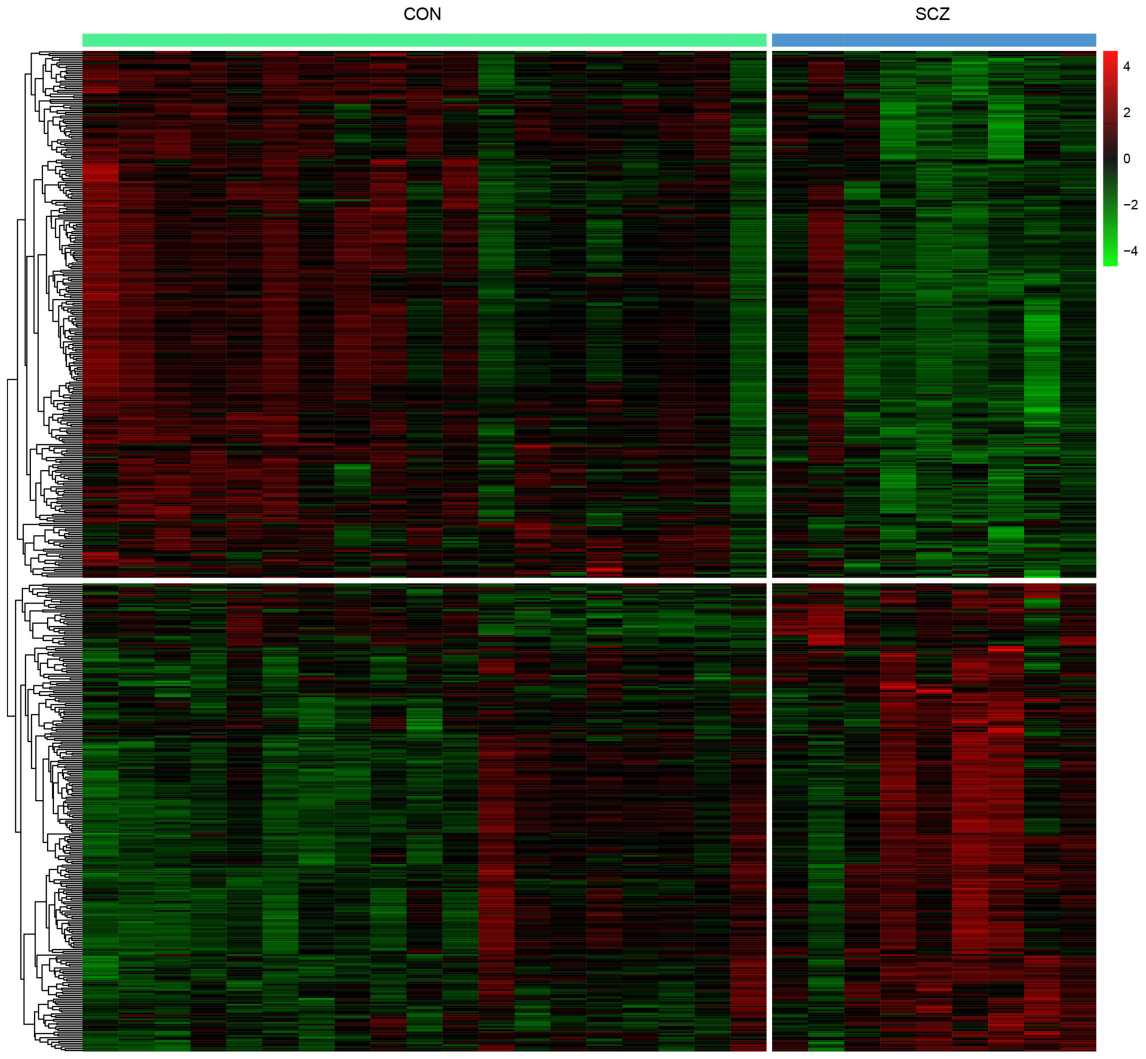
Heat map of DEGs in PBMCs.

Differential expression analysis was performed to elucidate the altered expression of PBMCs genes in patients with schizophrenia compared with healthy controls. A total of 542 DEGs were identified with 255 upregulated and 287 downregulated DEGs (Supplementary Table 3). The heat map indicates that patients with schizophrenia were discriminated from healthy controls by the expression levels of the DEGs (Figure 1).

**Figure 2:**
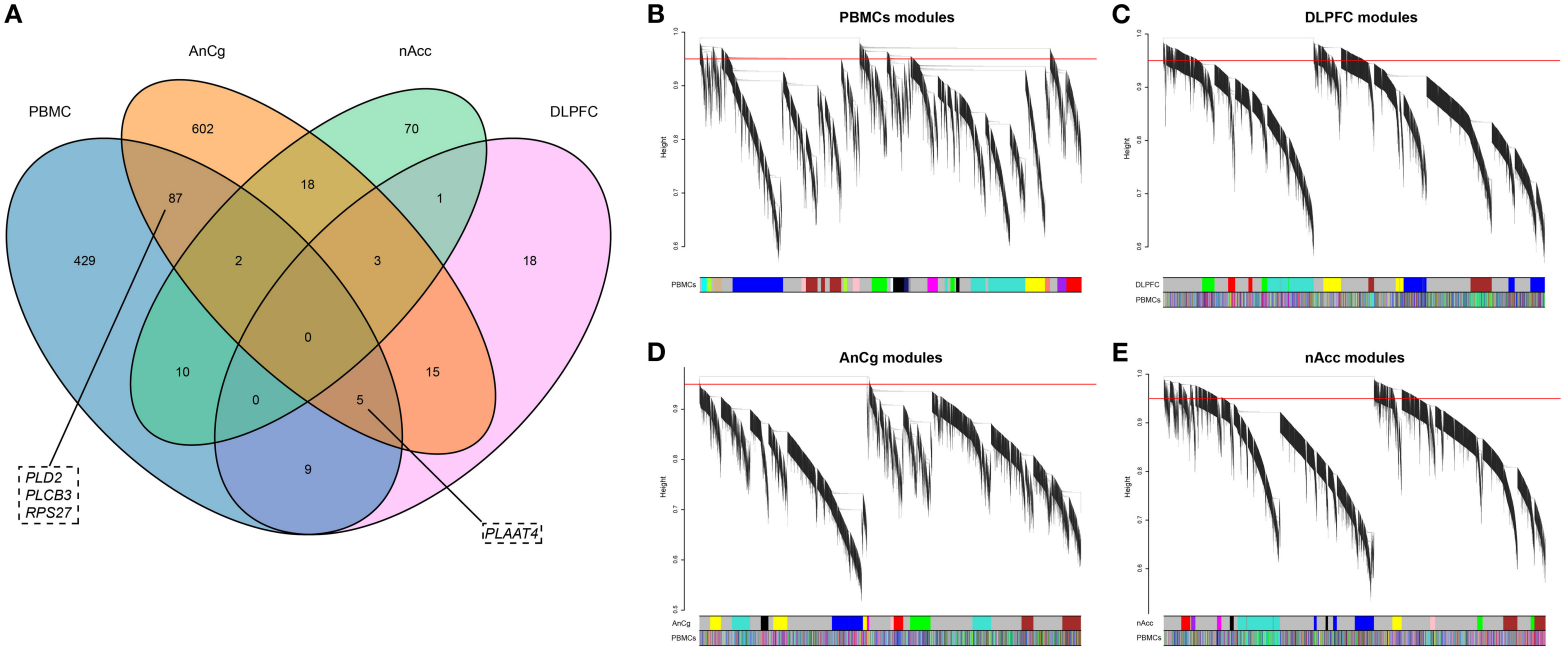
**(A)** The Venn diagram of the DEGs in PBMCs, DLPFC, AnCg, and nAcc. **(B–E)** Hierarchical clustering plots for these four data sets. The y-axis represents the network distance with values closer to zero illustrating greater similarity of gene expression, and the x-axis represents the gene modules in each data set. The PBMC module colors are mapped to each brain tissue module with the PBMC color panel denoting the degree of similarity.

To discern if there were identical DEGs between brain and PBMCs, DEGs in the three brain regions were identified. A total of 51, 732, and 104 DEGs were identified in DLPFC, AnCg, and nAcc, respectively. Among these DEGs, 840 genes were differentially expressed in at least one brain region (Supplementary Table 4), and 113 of these DEGs were also differentially expressed in PBMCs (Figure 2A).

### Similarity of Network Expression Patterns in PBMCs and the Brain

Although many overlapping DEGs were observed in both PBMCs and brain regions, it is still unknown whether the expression patterns between PBMCs and brain tissues were similar. To understand the correlation of gene expression patterns between PBMCs and brain tissues based on the gene networks, weighted gene co-expression networks were constructed in each data set. The soft thresholds of each data set are shown in Supplementary Figure 2, and the co-expression network of PBMCs is shown in Figure 2B.

**Figure 3:**
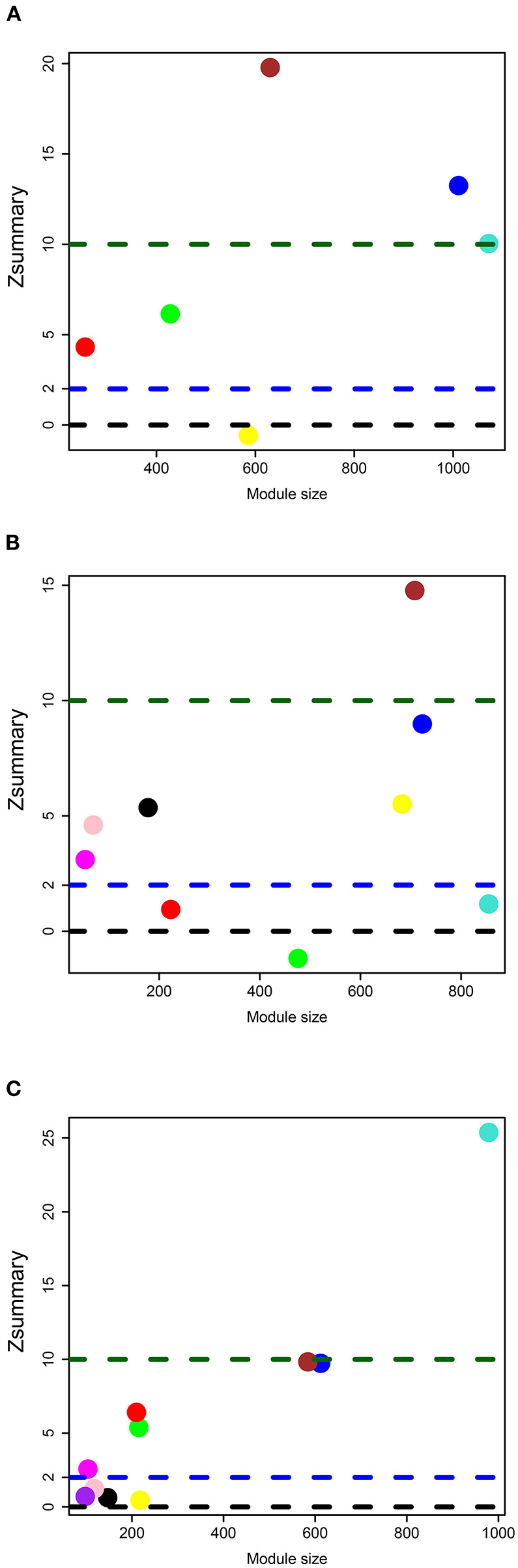
Module preservation statistics for **(A)** DLPFC, **(B)** AnCg, and **(C)** nAcc data sets. The y-axis represents *Zsummary*, and the x-axis represents the number of genes in each module. Each circle represents a different gene module in brain tissue, and the modules above the blue dotted line represent that it is preserved in PBMCs.

In the data set of DLPFC, we acquired six gene modules by WGCNA (Figure 2C), and the preserved degree of each module in the network of PBMCs was assessed by preservation statistics. Five out of these modules were preserved in PBMCs (*Zsummary* > 2; Figure 3A). The enrichment analysis of the DEGs in PBMCs exhibited that 92 DEGs in PBMCs overlapped with the blue module in brain tissue, and 91 DEGs in PBMCs overlapped with the brown module in brain tissue (FDR < 0.05) (Supplementary Tables 5, 6). The two sets of DEGs were defined as preserved DEG clusters and named DLPFC-blue and DLPFC-brown.

In the data set of AnCg, we obtained nine gene modules by WGCNA (Figure 2D). As shown in Figure 3B, six of these modules were preserved in PMBCs. There were 55 DEGs in PBMCs overlapped with the brown module in brain tissue, and 15 DEGs in PBMCs overlapped with the pink module in brain tissue (FDR < 0.05). They were named AnCg-brown and AnCg-pink clusters.

In the data set of nAcc, a total of 10 gene modules were acquired (Figure 2E), among which six modules were preserved in PBMCs (Figure 3C). The enrichment analysis of the DEGs in PBMCs showed that 127 DEGs in PBMCs overlapped with the turquoise module in brain tissue (FDR < 0.05). It was named the nAcc-turquoise cluster.

### Functional Analysis and Expression Validation of Preserved DEGs Clusters

To explore the shared changes of biological functions in the PBMCs and brain tissues of schizophrenia, the functional analyses of each preserved DEG cluster mentioned above were performed separately by IPA (Supplementary Table 7).

**Figure 4:**
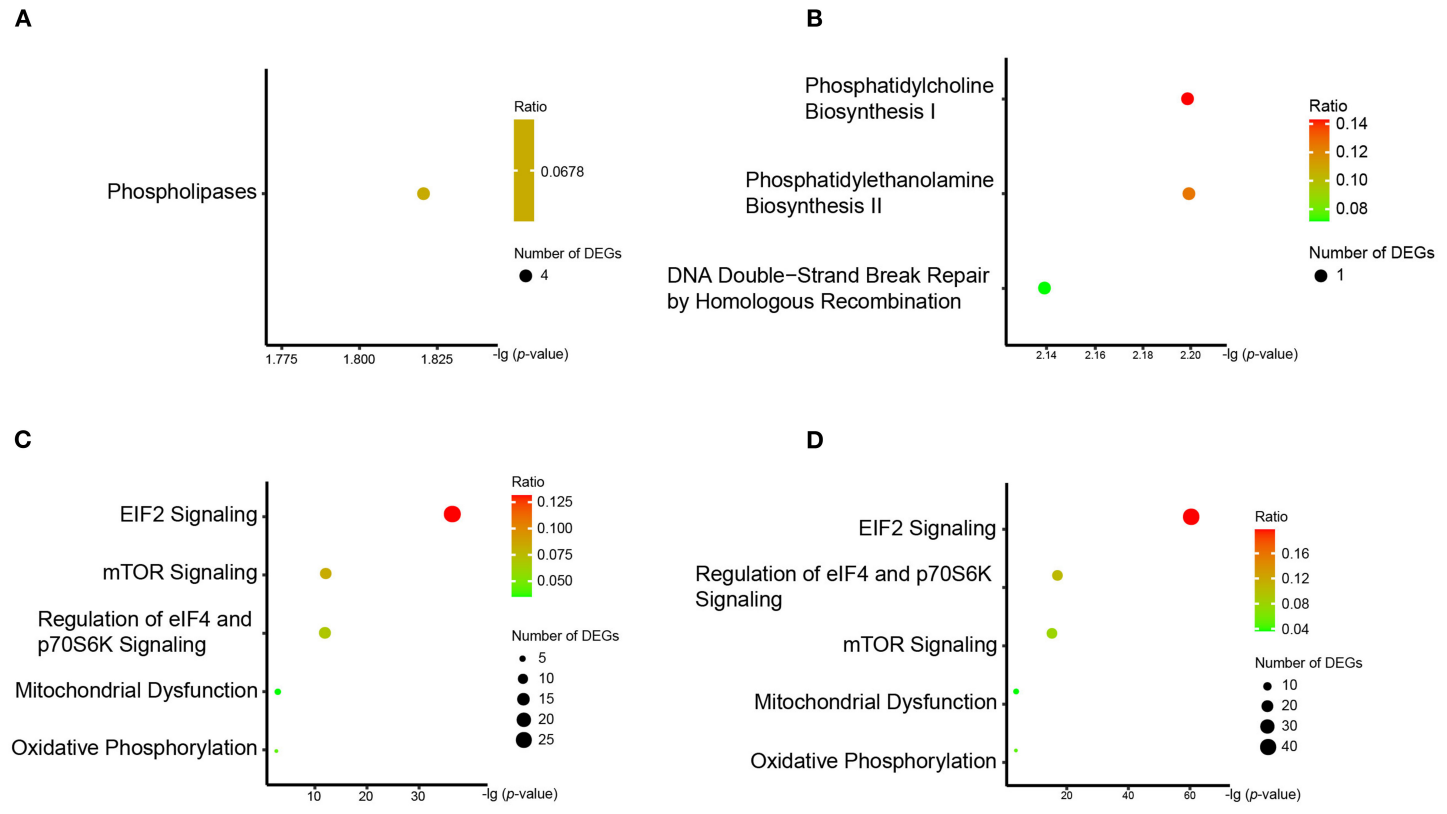
The results of IPA pathway analysis in **(A)** DLFC-blue, **(B)** AnCg-pink, **(C)** DLPFC-brown, and **(D)** nAcc-turquoise clusters. The y-axis represents all pathways enriched by the DEG cluster, and the x-axis represents the *p*-value after Benjamini–Hochberg correction.

**Figure 5:**
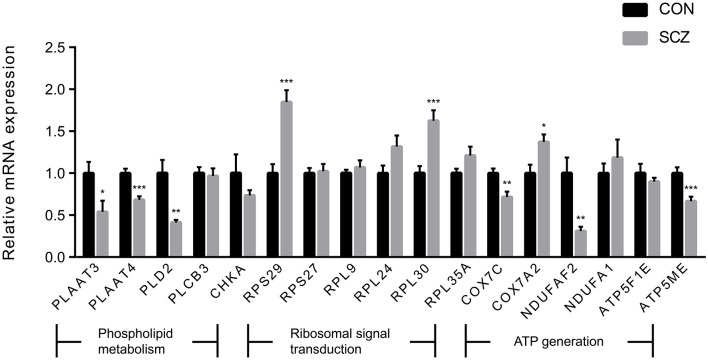
The expression levels of genes of interest. Values are exhibited as mean ± standard error of mean (n = 12 per group). Significant differences are denoted as follows: *p-value < 0.05, **p-value < 0.01, ***p-value < 0.001.

We observed three pathways associated with phospholipid metabolism in the enrichment analysis. The “phospholipases pathway,” which involved four DEGs (*PLD2, PLCB3, PLAAT3*, and *PLAAT4*), was enriched by the DLPFC-blue cluster (Figure 4A). “Phosphatidylcholine biosynthesis I” and “phosphatidylethanolamine biosynthesis II” were enriched by the cluster of AnCg-pink (Figure 4B) and referred to one DEG (*CHKA*). The results of qPCR validation are shown in Figure 5; the relative expression of *PLD2* (*t* = 3.712, *p* = 0.003), *PLAAT3* (*t* = 2.423, *p* = 0.024), and *PLAAT4* (*t* = 4.819, *p* < 0.001) displayed as significantly downregulated in patients with schizophrenia compared with healthy controls, and *PLCB3* (*t* = 0.306, *p* = 0.762) and *CHKA* (*t* = 1.142, *p* = 0.266) were found to have no significant difference.

We also found that another three pathways (“eIF2 signaling,” “mTOR signaling,” and “regulation of eIF4 and p70S6K signaling”) were enriched by numerous ribosomal protein genes. These pathways, which are related to ribosomal signal transduction, were enriched by the DLPFC-brown and nAcc-turquoise clusters (Figures 4C,D). There were 27, 13, and 12 DEGs involved in these three pathways on the enrichment analysis of the DLPFC-brown cluster, respectively. On the other hand, there were 42, 16, and 16 DEGs in the nAcc-turquoise cluster referred to these three pathways, respectively. All of these DEGs were ribosomal protein genes, and the top six genes with the largest FCs (*RPS29, RPS27, RPL9, RPL35A, RPL24, RPL30*) were selected for qPCR validation, among which *RPS29* (*t* = 4.741 *p* < .001) and *RPL30* (*t* = 4.115, *p* = < 0.001) were significantly different between the two groups, and *RPS27* (*t* = 0.198, *p* = 0.845), *RPL9* (*t* = 0.717, *p* = 0.481), *RPL35A* (*t* = 1.767, *p* = 0.091), and *RPL24* (*t* = 1.957, *p* = 0.063) were not statistically different between the two groups.

The DLPFC-brown and nAcc-turquoise clusters also enriched in two pathways (“mitochondrial dysfunction” and “oxidative phosphorylation”) that associated with energy metabolism (Figures 4C,D). In the DLPFC-brown cluster, there were six DEGs (*ATP5F1E, ATP5ME, COX7C, COX7A2, NDUFA1*, and *NDUFAF2*) taking part in the pathway of “mitochondrial dysfunction” and four DEGs (*ATP5F1E, ATP5ME, COX7C*, and *NDUFA1*) involved in the pathway of “oxidative phosphorylation.” Meanwhile, in the nAcc-turquoise cluster, a total of six DEGs (*ATP5F1E, ATP5ME, COX7C, COX7A2, NDUFA1*, and *NDUFAF2*) referred to “mitochondrial dysfunction” and five DEGs (*ATP5F1E, ATP5ME*, COX7C, *COX7A2*, and *NDUFA1*) involved in “oxidative phosphorylation.” As shown in Figure 5, the relative expression of *COX7C* (*t* = 3.318, *p* = 0.003), *COX7A2* (*t* = 2.747, *p* = 0.012), *ATP5ME* (*t* = 3.748, *p* < 0.001), and *NDUFAF2* (*t* = 3.547, *p* = 0.002) exhibited significant downregulation in patients with schizophrenia compared with healthy controls, and *ATP5F1E* (*t* = 0.828, *p* =.421) and *NDUFA1* (*t* = −0.739, *p* = 0.470) were not statistically different between the two groups.

The AnCg-brown cluster was not enriched in any pathway after correction. We noticed four of the DEGs (*PLD2, PLCB3, PLAAT4, RPS27*) involved in these pathways were also differentially expressed in the brain of patients with schizophrenia (Figure 2A). This provided supporting evidence for the dependability of the results of preservation analysis.

## Discussion

In this study, we detected mRNA expression alterations in PBMCs from patients with schizophrenia and healthy controls by transcriptomics analysis and combined brain tissue transcriptome public data sets for comprehensive bioinformatics analysis. We found the expression patterns of several pathways were similar in PBMCs and brain tissues of patients with schizophrenia. The DEGs of interest involved in these pathways were validated using qPCR. Our data further proved the dysfunction of schizophrenia in phospholipid metabolism, ribosomal signal transduction, and energy metabolism.

Our integrated analysis revealed a dysregulated phospholipid metabolism pathway in the patients with schizophrenia. The important effects of several types of phospholipases in the pathogenesis of schizophrenia have been confirmed. Smesny et al. (24) found activity of phospholipase A2 increased in patients with schizophrenia and correlated with the severity of negative symptoms. PLC-β1 knockout mice exhibited rodent schizophrenia-like phenotypes, such as hyperactivity and cognitive impairment (25). However, the relation between phospholipase D (PLD) and schizophrenia is still unclear. Phosphatidic acid as a hydrolysate of the PLD catalytic reaction is also an important intracellular second messenger that activates Raf-1, mTOR, and other signaling proteins and is involved in a series of biological processes (26–28). This may be access by which PLD participates in the pathogenesis of schizophrenia. Besides this, phospholipase is also involved in the process of phospholipid metabolism *in vivo*. A dysregulated phospholipid metabolism pathway has been uncovered in a study using microarray public data in brain tissues of patients with schizophrenia (29). This result, which is consistent with ours, implies dysfunctional phospholipid metabolism is closely related to schizophrenia. A magnetic resonance spectroscopy metabolomics study reported decreased synthesis of membrane phospholipids in the prefrontal cortex of children and adolescents at high risk for schizophrenia and supplied a clue for the forecast of schizophrenia (30). Our previous study, which was based on liquid chromatography/mass spectrometry technology, found altered phosphatidylcholine and phosphatidylethanolamine in the plasma of patients with schizophrenia (31). Researchers observed that the content of phosphatidylcholine decreased in the brain and the apoptosis of hippocampal neurons increased after a low-choline diet for rats (32). This evidence indicates that there is dysregulated phospholipid metabolism present in both brain tissue and peripheral blood of schizophrenia, and altered peripheral phospholipid levels could affect the function of the CNS.

In the present study, we found that three dysfunctional pathways (“eIF2 signaling,” “mTOR signaling,” and “regulation of eIF4 and p70S6K signaling”) were highly related to mRNA translation and protein synthesis, and almost all of the ribosomal protein genes involved in these three signaling pathways were downregulated in schizophrenia. Protein synthesis hypofunction was also observed in schizophrenia patient–derived olfactory cells (33) and in the postmortem prefrontal cortex of patients with schizophrenia (34). However, Hori et al. (35) argues that RPL17 and RPL34 were upregulated in the blood of patients with schizophrenia. Although the changes in ribosomal protein genes are not the same in these findings, ribosomal protein dysfunction, which is widely observed in schizophrenia, indicates that dysfunctional protein synthesis is highly associated with the etiology of schizophrenia. Additionally, there a review suggested protein synthesis was arrested via inhibition of eIF2B by schizophrenia-related stressors (36). Kim et al. (37) proved dendritic development of newborn neurons regulated by disrupted-in-schizophrenia one during adult hippocampal neurogenesis needed the participation of the AKT-mTOR pathway. The regulation of risk genes of schizophrenia on neurodevelopment might be accomplished by convergence in the AKT/mTOR pathway (38). Studies using the MK-801 (a selective and non-competitive NMDAR antagonist) neurodevelopmental animal model of schizophrenia found an altered p70S6K-S6/eIF4B pathway and protein translation in the frontal cortex of schizophrenic-like rat (39, 40). These findings support our results on the dysfunction of these three signaling pathways.

We also found that dysfunctional mitochondrial and oxidative phosphorylation were associated with schizophrenia. Glausier et al. (41) suggests 41% of mitochondrial-related genes were differentially expressed in the DLPFC of patients with schizophrenia. Prabakaran et al. (7) also found that genes related to mitochondrial dysfunction and oxidative stress could distinguish patients with schizophrenia from healthy controls. Additionally, we found ATP5ME and NDUFAF2 were differentially expressed. ATP5ME encodes the e subunit of Fo complex that constitutes the membrane-spanning component of adenosine-triphosphate synthase (42), and the encoding product of NDUFAF2 is the one of the components of complex I in the electron transport chain (43). These results are accordant to impaired complex I, II, and IV in the oxidative phosphorylation system (44, 45) and reduced activity of complex in the mitochondrial electron transport chain (46, 47) observed by other researchers in patients with schizophrenia. In our previous metabolomics study, the changes in metabolites related to energy metabolism were also observed in PBMCs of patients with schizophrenia by gas chromatography mass spectrometry (48). Our results demonstrate that disturbance of energy metabolism occurs at both the metabolic and transcriptional levels in patients with schizophrenia.

There are several limitations to consider in interpreting results from this study. First, the small sample size had limited statistical efficacy, and it is necessary to increase the number of samples to verify the present results in the later work. Second, these healthy controls met the inclusion and exclusion criteria, but they were not assessed by General Health Questionnaire (GHQ) in our study. Third, the changes of crucial regulators in the protein level, which are related to phospholipid metabolism, ribosome signal transduction, and energy metabolism, needed further evaluation by the Western blot assay. Last, we identified shared pathways in PBMCs and brain tissue by correlation analysis, but the specific bidirectional regulatory relationship between them is still unclear. Experiments with cerebral stereotaxic injection for a schizophrenic-like animal model are necessary in future research.

## Conclusions

In this study, we found expression patterns of several pathways to be similar in PBMCs and brain tissue of patients with schizophrenia. The characteristics of these pathways afforded supportive proof for dysfunction of phospholipid metabolism, ribosome signal transduction, and energy metabolism in schizophrenia. On the basis of the analysis of single DEGs, the further addition of cross-tissues co-expression network analysis may provide novel insight for elucidating the underlying molecular basis of schizophrenia and other psychiatric disorders. Meanwhile, this approach may contribute to discovering new peripheral blood gene biomarkers of schizophrenia.

## Data Availability Statement

The original contributions presented in the study are publicly available. This data can be found here: NCBI BioProject Accession PRJNA735935.

## Ethics Statement

The studies involving human participants were reviewed and approved by the Ethical Committee of Chongqing Medical University. The patients/participants provided their written informed consent to participate in this study.

## Author Contributions

PX and XS designed the study, interpreted the results, and wrote the original draft. PX and YL supervised the whole process. XS, JP, and SG completed the data preprocessing and analysis. XS, YL, JP, XZ, XC, WC, HW, and LZ collected participants. XC, YC, and KC analysis the plasma samples. All authors contributed to the article and approved the submitted version.

## Funding

This work was supported by the National Key Research and Development Program of China (2017YFA0505700), the Non-profit Central Research Institute Fund of Chinese Academy of Medical Sciences (2019PT320002), the Natural Science Foundation Project of China (81820108015), and the China Postdoctoral Science Foundation (2020M673160).

## Conflict of Interest

The authors declare that the research was conducted in the absence of any commercial or financial relationships that could be construed as a potential conflict of interest.

## Publisher's Note

All claims expressed in this article are solely those of the authors and do not necessarily represent those of their affiliated organizations, or those of the publisher, the editors and the reviewers. Any product that may be evaluated in this article, or claim that may be made by its manufacturer, is not guaranteed or endorsed by the publisher.
