# Supplementary figures and images for: Transcriptomics Analysis Reveals Shared Pathways in Peripheral Blood Mononuclear Cells and Brain Tissues of Patients With Schizophrenia
Source: Front Psychiatry. 2021 Sep 22;12:716722. doi: 10.3389/fpsyt.2021.716722 (PMC8492981; doi:10.3389/fpsyt.2021.716722)

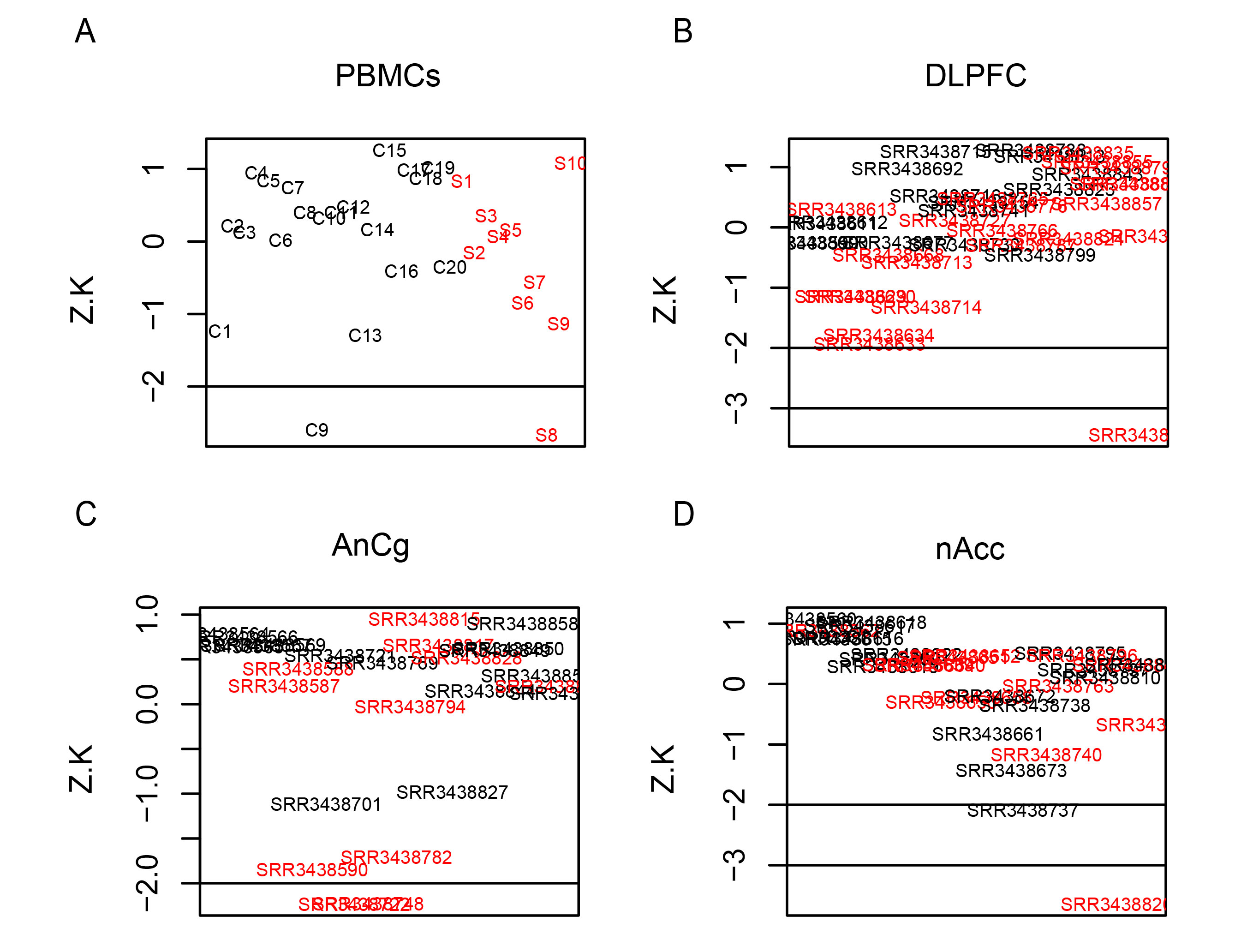

Supplement: Supplementary file 1 [file Image_1.JPEG]

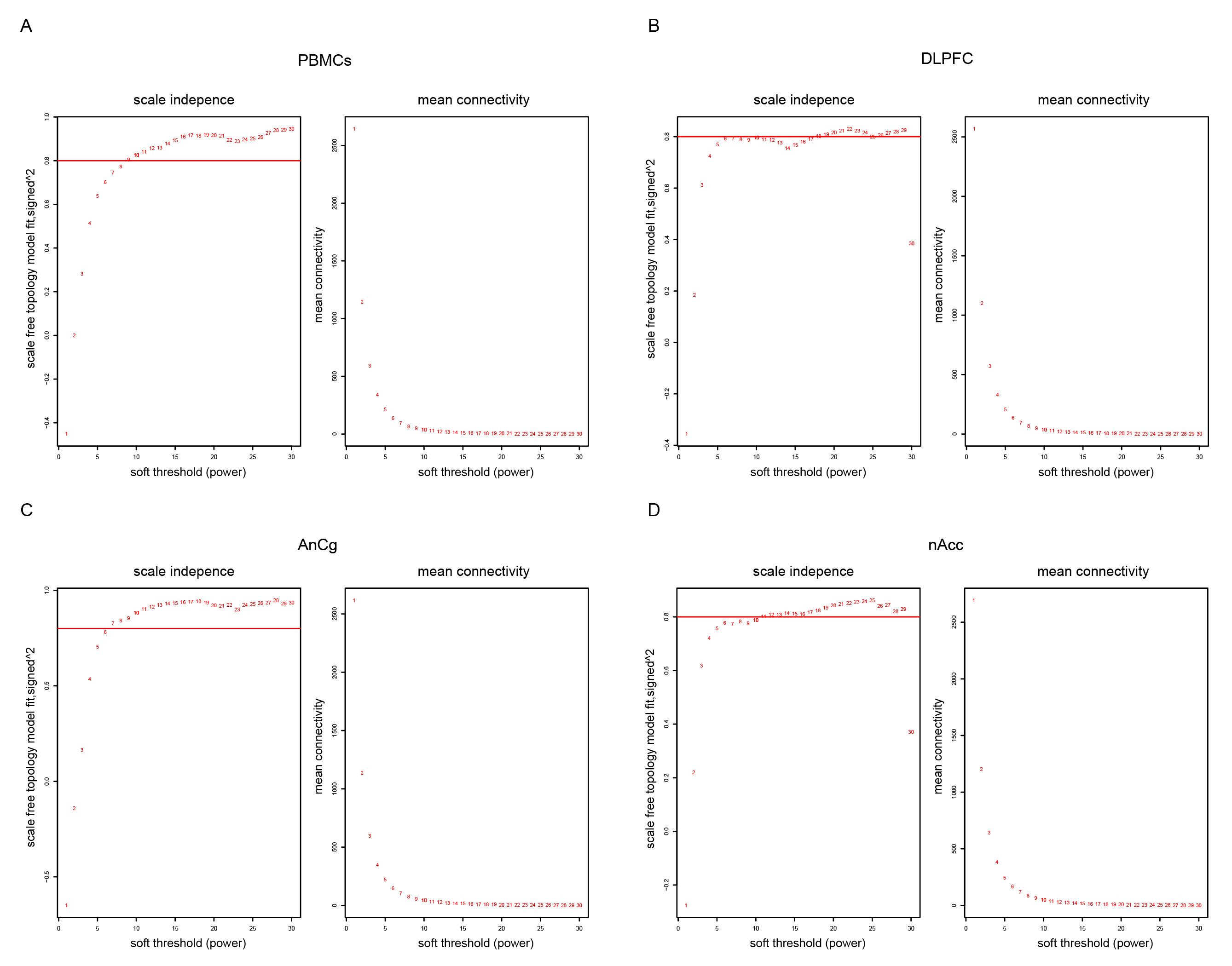

Supplement: Supplementary file 2 [file Image_2.JPEG]
